# Impact of the COVID-19 Pandemic on Postpartum Depression and Length of Stay in a Large Academic Center in New Jersey

**DOI:** 10.7759/cureus.84185

**Published:** 2025-05-15

**Authors:** Kanchi Chadha, Ethan Slouha, Antonia Oladipo

**Affiliations:** 1 Obstetrics and Gynecology, Hackensack University Medical Center, Hackensack, USA; 2 Obstetrics and Gynecology, St. George's University School of Medicine, St. George's, GRD

**Keywords:** covid 19, edinburgh postnatal depression scale, gynaecology and obstetrics, perinatal psychiatry, postpartum depression

## Abstract

Introduction: Postpartum depression (PPD) is screened by the Edinburgh Postnatal Depression Scale (EPDS), where a score of 10 - 12 indicates possibly having PPD and a score of ≥ 13 indicates PPD. This publication aims to describe the impact of the COVID-19 pandemic on the hospital length of stay (LOS) of parturients following a PPD diagnosis.

Method: The CDC identified five peak months of COVID-19 cases in NJ between January 2020 and March 2022; 10,967 parturients at Hackensack University Medical Center (HUMC) filled out the EPDS after delivery and were split into two groups based on their EPDS scores: 10-12 and ≥ 13. Groups were then correlated with the five peak months, assessing the LOS via the t-score and one-way ANOVA.

Results: Across the five peak months, 71 (3.67%) were found to have an EDPS of ≥ 10, with 71.8% in group 1 and 28.2% in group 2. In these months, the average LOS was 2.2 days, significantly less than the LOS in patients with an EDPS of ≥ 10 (p = 0.03). The average LOS in groups 1 and 2 was 2.57 and 2.4 days, respectively, with no significant difference.

Conclusion: The difference in the LOS, not accounting for extenuating factors, means that individuals with possible or diagnosed PPD had a longer stay, which may be associated with proper psychiatric care being employed prior to discharge. Further research assessing the LOS for PPD following COVID-19 may elucidate whether proper care is still being given for PPD following delivery.

## Introduction

Parturients typically feel many emotions following the birth of their child, from pleasure and joy to bouts of crying and sadness, coined postpartum blues, but escalation and intensification of these symptoms lead to the diagnosis of postpartum depression (PPD) [1]. PPD affects 13-25% of parturients and persists longer and with debilitating symptoms, ultimately affecting the parturient’s relationship with their baby [1,2]. PPD presents with a wide range of symptoms that can lead to a misdiagnosis, including struggling with everyday activities, fatigue, sadness, insomnia, and anhedonia [3]. The pathophysiology of PPD is still up for debate, and the leading hypotheses are due to the alterations in the hypothalamic-pituitary-adrenal axis, gamma-aminobutyric acid type A receptors, and lactogenic hormones [1,3,4]. PPD is screened for using the Edinburgh Postnatal Depression Scale (EPDS), which is a 10-item questionnaire in which a score of 10 or greater indicates possible PPD, and a score of 13 or greater indicates the patient has PPD [1,4-7]. PPD can lead to breastfeeding failure, marital discord, poor maternal-infant bonds, harmful parenting practices, and less favorable outcomes concerning the child’s psychological and physical development [1].

The COVID-19 pandemic has been traced worldwide and has affected many hospitals’ treatment and care practices, including labor and delivery and psychiatric management. COVID-19 has been observed to significantly affect individuals in vulnerable populations, such as pregnant and delivering women [8]. Studies focusing on the PPD rate and care of parturients who had just delivered are scarce, but it was found that parturients experienced significantly more stress levels during the pregnancy and birth process, stress specifically associated with contracting COVID-19 due to the associated morbidity and mortality [8-10]. Some studies observed that PPD was significantly higher during COVID-19, with over 27% and up to 54.2% developing PPD during the postpartum period possibly due to social isolation, limited interaction with families, and possibly shorter hospital stays [10-12]. Usually, parturients experiencing any psychiatric conditions following the birth of their child typically have a more extended hospital stay from admission to discharge than healthy parturients, averaging up to 8.13 days in one study [13]. However, due to the constraints on hospital stay in the maternity wards during COVID-19, the length of stay (LOS) in the hospital may be decreased significantly in parturients with an EPDS score of 10 or greater compared to pre-COVID-19 times. Still, it’s not clear whether this level of care was reproducible during the pandemic.

This study aimed to examine EPDS scores and the LOS from delivery to discharge in parturients possibly suffering from and having PPD during peak points in the epidemic and assess its impact on maternal care.

## Materials and methods

Study population

Study subjects included all primiparous women aged 15-53 who gave birth in Hackensack University Medical Center between January 2020 and March 2022: April 2020, July 2020, January 2021, September 2021, and January 2022, which were peak COVID-19 times; 10,967 female patients delivered infants during the peak times assessed. Participant characteristics such as age, race, and ethnicity were self-reported by the participants according to CDC’s classification.

Study design

This retrospective cohort study used the CDC database to determine peak times during the COVID-19 pandemic in NJ between January 2020 and March 2022. It is a standardized practice that all female patients who give birth at HUMC fill out the EPDS following the birthing process to determine the risk for PPD. The EPDS scores from parturients who gave birth during the peak COVID-19 time were then grouped into EPDS < 10, EPDS 10-12, and EPDS greater than 13. The records of parturients giving birth, their EPDS score, and the peak COVID-19 times were linked and cross-examined. The LOS for parturients in both groups was acquired and then compared to determine if there was significance.

Definitions

The peak COVID-19 times were defined as periods of significant increase in COVID-19 in NJ during the COVID-19 pandemic. Postpartum is demarcated as the time frame starting from the day of birth to 365 days later. The principal diagnosis was PPD based on a positive EPDS greater than 13. Parturients were split into two groups based on their EPDS score: group 1: EPDS 10 - 12 and group 2: EPDS ≥ 13. The LOS per billing code is the total number of days from hospital admission until discharge; however, this paper defines LOS as the day of delivery to discharge.

Statistical analysis

The average EPDS scores for each peak in COVID-19 were analyzed through one-way ANOVA to compare differences between each peak using Excel (Microsoft Corporation, Redmond, USA). Then, parturients were grouped into groups 1 and 2, and the differences were compared based on the number of parturients in each group, and significance was determined. The LOS of group 1 and that of group 2 were also compared using a t-score to determine if the difference was significant.

Ethics approval

The authors assert that all procedures contributing to this work comply with the ethical standards of the relevant national and institutional committees on human experimentation and with the Helsinki Declaration of 1975, as revised in 2013. All procedures involving human subjects/patients were approved by the IRB Board at HUMC, NJ, USA, # Pro2023-0229.

## Results

During the period January 2020 to March 2022, 10,967 women were admitted to Labor & Delivery (L&D) to deliver their child. Of those 10,967 women, 433 (3.95%) had an EPDS of 10 or greater, with an average age of 32.46. In the peak COVID month identified, 1933 women were admitted to L&D, and within this timeframe, 71 (3.67%) were found to have an EPDS of 10 or greater, with an average age of 32.58 (SD 5.01). These parturients were then split into two groups. Group 1 consisted of 51 (71.8%) parturients with an average age of 32.45 (SD 5.03), and group 2 consisted of 20 (28.2%) parturients with an average age of 32.5 (SD 5.09). In consideration of influential factors, demographics (race, ethnicity, gravidarum, parity, vaginal delivery, and cesarean section) are displayed in Table 1 with no significant difference in demographics.

**Table 1:** Baseline Characteristics and Demographics of the Population Numbers presented as n(%) or mean (SD). EPDS: Edinburgh Postnatal Depression Scale

	5 COVID Peaks (n = 2025)	EPDS 10+ (n = 71)	Group 1 (EPDS 10-12) (n = 51)	Group 2 (EPDS 13/greater) (n = 20)
Average Age	32.6 (5.18)	32.46 (5.01)	32.45 (5)	32.5 (5.1)
Ethnicity				
Non-Hispanic	67.41%	66.20%	64.71%	70%
Hispanic	29.18%	26.76%	29.41%	20%
Unknown	2.38%	4.23%	1.96%	10%
Declined	1.03%	2.82%	3.92%	0%
Race				
American Indian/Alaska Native	0%	0%	0%	0%
Asian	17.63%	23.95%	23.52%	25%
Korean	5.07%	7.04%	7.84%	5%
Vietnamese	0.10%	1.41%	0%	5%
Asian Indian	6.62%	9.86%	9.80%	10%
Chinese	1.14%	1.41%	0%	5%
Japanese	0.41%	0%	0%	0%
Other Asian	4.29%	4.23%	5.88%	0%
African American	5.38%	5.63%	5.88%	5%
Native Hawaiian or Other Pacific Islander	1.24%	2.82%	1.96%	5%
Caucasian	36.73%	18.31%	21.57%	10%
Other	35.44%	42.25%	41.18%	45%
Unknown	2.69%	5.63%	3.92	10%
Declined	0.88%	1.41%	1.96%	0%
Gravidum	2.65 (1.76)	2.48 (1.67)	2.49 (1.67)	1.64 (1.74)
Parity	2.06 (1.36)	1.87 (1.05)	1.9 (1.1)	1.8 (1.04)
C-Section	33.47%	39.44%	43.14%	30%
Vaginal	66.53%	60.56%	56.86%	70%

The LOS was then evaluated regarding the EPDS score, and the average LOS during the five peak COVID months was 2.18 days (SD 0.04) with those who had an EPDS < 10, significantly less than the LOS in parturients with an EPDS of 10 or greater which was 2.52 (SD 0.28; t-value = 1.96, p = 0.02, 95% CI [0.05, 0.63]). The average LOS in group 1 overall was 2.57 (SD 1.31) days, whereas group 2’s average LOS was 2.4 (SD 0.75) days, with no significant difference observed (t-value = 1.96, p = .53, 95% CI [-0.35, .07]). In either group, there was no difference between the months used. Individual months of the peak COVID times were compared between group 1 and group 2, and there was no significant difference between the groups in any months seen in Table 2.

**Table 2:** Results from Analysis Numbers presented as n(%) or mean(SD). EPDS:  Edinburgh Postnatal Depression Scale; LOS: length of stay

EPDS/LOS						
Peak COVID	Apr-20	Jul-20	Jan-21	Sep-21	Jan-22	Combined
Total Charts	383	433	381	435	393	2025
10+ EPDS (%)	18 (4.71%)	14 (3.23%)	10 (2.62%)	16 (3.68%)	15 (3.82%)	73 (3.6%)
10-12 EPDS (%)	15 (3.92%)	10 (2.31%)	7 (1.83%)	11 (2.53%)	9 (2.29%)	52 (2.57%)
13+ EPDS (%)	3 (0.78%)	4 (0.92%)	3 (0.79%)	5 (1.15%)	6 (1.53%)	21 (1.04%)
Overall LOS	2.13 (0.92)	2.27 (0.81)	2.16 (0.89)	2.24 (0.8)	2.17 (0.92)	2.2 (0.87)
10 or greater EPDS LOS	2.38 (0.92)	2.15 (0.8)	2.11 (0.33)	2.65 (0.72)	3.13 (2)	2.52 (1.18)
10-12 EPDS LOS	2.47 (1)	2.1 (0.57)	2.17 (0.41)	2.55 (0.69)	3.56 (2.55)	2.57 (1.32)
13+ EPDS LOS	2 (0)	2.33 (1.53)	2 (0)	2.8 (0.84)	2.5 (0.55)	2.4 (0.75)

## Discussion

With a parturient undergoing tremendous strain and physiological changes during pregnancy and the actual delivery process, they are already susceptible to psychiatric conditions like PPD [1,2]. Stressors such as planning for the infant, worrying about the health of the infant, financial needs, antenatal depression, antenatal anxiety, and others have already been attributed to increasing the chance of PPD [14,15]. The most recent estimate of PPD before COVID was that an average of 25% of parturients developed PPD [1,2]. This study found that 433 (3.8%) of parturients between January 2020 and March 2022 during the COVID-19 pandemic were at risk or had PPD within 24 hours after giving birth. Narrowing our scope down to peak months during the COVID period, we observed that 71 (3.67%) of parturients had an EPDS of 10 or greater, but only 20 out of the 71 were diagnosed with PPD, having an EPDS of 13 or greater.

Other smaller-scale studies have found that up to 38.2% of parturients screened positive for PPD during the COVID-19 pandemic within weeks of giving birth [13]. These changes are paired with higher scores for depression and anhedonia in the COVID-19 study groups, suggesting that while pregnant women are normally considered a vulnerable population, during COVID-19, they were an especially vulnerable population that requires more attention [15]. COVID-19 adds another layer of stress as the parturients now have to worry about not only themselves being infected with COVID-19 but also their newborns as well [9]. Another stress for parturients is the limited visitor and visitation policies instated during the pandemic, as more extensive support networks may have comforted some parturients prior to the pandemic. It is important to note that in both the pre-COVID-19 group and the COVID-19 group in one study, factors such as social support and satisfaction with birth were significantly associated with a decreased risk of PPD [14]. Not only is COVID-19 suspected to intensify the prevalence of PPD, but it also created barriers to accessing the appropriate resources and healthcare needs, either through limited visitation appointments for specialists or even barriers set by the government, such as the stay-at-home rules [11].

The duration of hospital stay for parturients prior to the pandemic was relatively short at an average of two days, which seemed to continue during the pandemic with no significant changes [16]. In our study, it was found that this was still less than what we found for parturients at risk or diagnosed with PPD. Prior to COVID-19, parturients had relatively easy access to psychiatric care immediately following birth and weeks following birth if they chose or were urged to seek help, as there were no barriers to access, such as high patient load or lack of face-to-face interaction [17]. The help acquired immediately following birth typically contributed to a longer LOS in the hospital to ensure they were fit to go home [17]. We found that compared to the 1933 female patients who delivered during the five peaks of COVID months, parturients with an EPDS of 10 or greater had a significantly longer LOS. It’s unclear whether this is due to extra care or extenuating circumstances such as maternal or obstetric complications, as this study did not evaluate these factors. Comparing group 1 and group 2, there was no significant difference in the LOS; in fact, group 1 had a slightly longer but insignificant LOS, which may be due to extenuating circumstances not identified. The LOS overall for parturients who had just given birth, vaginal or C-section, was significantly decreased during the pandemic, which is in line with what was observed in this study as the overall length of stay was slightly decreased compared to the estimated average of two days [12,17]. The LOS may have been greatly impacted by the early discharge initiatives to prevent the infectious spread of COVID-19 to the parturient and the infant. However, the early discharge initiatives could also provide early outpatient referral initiatives to reduce the risk of PPD to the parturient and child if they choose or are urged to follow.

Limitations of this study include several key factors. One major limitation is the strict population of the participants, which centered primarily in Bergen County, NJ, whereas using NJ as a whole would allow for more specific data. Another limitation is the number of individuals who identified as other Races, comprising over 1/3rd of our participants. Overall, though, the race distribution mostly met the defined races in Bergen County, NJ, aside from a decrease in the percentage of African Americans.

## Conclusions

This study observed no significant correlation between the five distinct peaks of COVID-19 in NJ and the EPDS screening results of PPD in parturients who had just given birth. There was a significant difference in the LOS when comparing parturients with EPDS scores < 10 and EPDS scores 10 or greater; however, there was no significant difference between groups 1 (EPDS 10-12) and 2 (EPDS greater than 13).
